# Oxidative Stress Produced by Hyperthyroidism Status Induces the Antioxidant Enzyme Transcription through the Activation of the Nrf-2 Factor in Lymphoid Tissues of Balb/c Mice

**DOI:** 10.1155/2019/7471890

**Published:** 2019-06-02

**Authors:** Melisa Costilla, Rodrigo Macri Delbono, Alicia Klecha, Graciela Alicia Cremaschi, María Laura Barreiro Arcos

**Affiliations:** ^1^Instituto de Investigaciones Biomédicas (BIOMED), Facultad de Ciencias Médicas, Pontificia Universidad Católica Argentina (UCA), Consejo Nacional de Investigaciones Científicas y Técnicas (CONICET), Buenos Aires, Argentina; ^2^Laboratorio de Radioisótopos, Facultad de Farmacia y Bioquímica (FFyB), UBA, Buenos Aires, Argentina; ^3^Departamento de Química Biológica, Facultad de Ciencias Exactas y Naturales (FCEyN), Universidad de Buenos Aires (UBA), Buenos Aires, Argentina

## Abstract

Hyperthyroidism is an endocrine disorder characterized by excessive secretion of thyroid hormones T3 and T4. Thyroid hormones exert pleiotropic actions on numerous tissues and induce an overall increase in metabolism, with an increase in energy demand and oxygen consumption. Therefore, the purpose of this study was to investigate the effects of hyperthyroidism on the production of reactive oxygen species (ROS) in lymph node and spleen cells of euthyroid and hyperthyroid mice, analyzing antioxidant mechanisms involved in the restitution of the cellular redox state. For this, thirty female Balb/c (H-2d) mice were randomly divided into two groups: euthyroid (by treatment with placebo) and hyperthyroid (by treatment with 12 mg/l of T4 in drinking water for 30 days). We found a significant increase in ROS and an increase in the genomic and protein expression of the antioxidant enzymes catalase (CAT) and glutathione peroxidase-1 (GPx-1) in lymph node and spleen cells of hyperthyroid mice. *In vitro* treatment with H_2_O_2_ (250 *μ*M) of the lymphoid cells of euthyroid mice increased the expression levels of CAT and GPx-1. The hyperthyroidism increased the phosphorylation levels of Nrf2 (nuclear factor erythroid 2-related factor) and the kinase activity of protein kinase C (PKC) and extracellular signal-regulated kinase (ERK). Additionally, we found an increase in the expression of the classic isoenzymes of PKC*α*, *β* and *γ*. In conclusion, these results indicated that the increase in ROS found in the hyperthyroid state induces the antioxidant enzyme transcription through the activation of the Nrf-2 factor in lymphoid tissues. This shows the influence of hyperthyroidism on the regulation of the cellular antioxidant system.

## 1. Introduction

Hyperthyroidism is an endocrine disorder characterized by an excess of function of the thyroid gland, which secretes high levels of thyroxine (T4) and triiodothyronine (T3) into the blood. The excess of thyroid hormones (TH) suppresses the production of thyrotropin (TSH) in the pituitary; therefore, the serum concentrations of this hormone are lower than normal.

TH are involved in the regulation of metabolism, differentiation, survival, and cell proliferation. They act through their interaction with nuclear receptors, producing the transcription of specific genes or, through their interaction with membrane receptors, triggering nongenomic effects [1]. In the lymphoid tissue, TH increases the proliferative response of T and B lymphocyte cells after an antigenic challenge and increases the humoral response with the production of specific antibodies [2]. It has been demonstrated *in vitro* that TH induce the proliferation of several lines of murine and human T-lymphomas. In addition, hyperthyroidism increases angiogenesis and tumor proliferation in mice inoculated with T lymphoma lines, which leads to a shorter survival of the mice [3–5].

TH induced an overall increase in metabolism that affects almost all tissues [6]. The increase in metabolic demand requires the synthesis of chemical energy or ATP through a set of oxidation-reduction reactions carried out by the mitochondria. The enzymatic complexes of the respiratory chain generate superoxide anion, a very reactive molecule that can dismute hydrogen peroxide; this compound in turn can react with transition metals to form hydroxyl ions with a high oxidation capacity. Due to its primary function of chemical energy production, mitochondria are considered the largest producer of reactive oxygen species (ROS) [7]. These observations are consistent with the increase in oxidative stress found in different tissues in the hyperthyroid state [8]. Additionally, the presence of T3 receptors in mitochondria that regulate mitochondrial activity and cellular metabolism has been demonstrated [9].

ROS participate in cell physiology regulating cell communication, substance transport, and gene transcription. However, the overproduction of reactive species produces an imbalance of the cellular redox state that can be harmful to the cells. ROS can react indiscriminately with various biomolecules such as the double bonds of lipids, the cysteine and methionine residues of proteins, and the C8 position in the deoxyguanosine of the DNA structure, thus leading to arrest of the cell cycle or to the cell apoptosis [10]. It has been described in tumor T lymphocytes that TH induce *in vitro* an increase in cell proliferation and limited production of nitric oxide and ROS; however, the prolonged treatment of these cells with TH induces an exacerbated increase in ROS and cellular apoptosis. Physiological concentrations of ROS could stimulate lymphocyte proliferative responses, while excessive amounts of ROS could be cytotoxic, leading to apoptosis [11].

The cellular redox state is regulated by a complex antioxidant system that prevents the toxic effect of ROS on the cells. The nonenzymatic antioxidant system is composed of a series of low molecular weight compounds that neutralize ROS, such as reduced glutathione (GSH), *α*-tocopherol, ascorbic acid, and coenzyme Q. The enzymatic antioxidant system consists of three main enzymes, superoxide dismutase (SOD), catalase (CAT), and glutathione peroxidase (GPx), which selectively neutralize the action of free radicals. Other enzymes such as glutathione reductase, glutathione-S-transferase, and glutamyl cysteinyl synthetase, without being strictly antioxidant enzymes, collaborate indirectly with GPx, since they contribute to regulating the intracellular pool of GSH, one of the main nonenzymatic cellular antioxidants. The SOD enzyme catalyzes the destruction of the superoxide anion radical, forming hydrogen peroxide that can be neutralized by the CAT and GPx enzymes. GPx is the most important enzyme to eliminate peroxides in the cells of mammals; it needs GSH as a cofactor, which is oxidized in the presence of free radicals [12].

Nrf-2 (nuclear factor erythroid 2-related factor 2) is a transcription activator which plays a key role in protecting the cell from oxidative stress and inflammation. It is primarily localized in the cytoplasm under normal conditions, but under conditions of oxidative stress, Nrf-2 migrates to the nucleus to upregulate the expression of genes with ARE (antioxidant response elements) in the promoter region. The upregulated genes include those involved in cellular protection, drug transport, detoxification, and antioxidant defense. Nrf-2 protein is expressed in various tissues, such as kidney, muscle, lung, heart, liver, and brain [13].

There are reports that the thyroid state modulates the expression of antioxidant enzymes in various tissues [14–16]. However, the effect of TH on the regulation of the expression of antioxidant enzymes and the cellular redox state in the lymphoid tissue has not yet been clarified. Therefore, our interest was to analyze the effects of hyperthyroidism on the production of ROS in lymphoid tissue cells, analyzing the molecular mechanisms involved in the regulation of the cellular redox balance. The study of the transduction signals that regulate the antioxidant system could be useful to identify molecular targets for the treatment of other pathologies that present high levels of oxidative stress.

## 2. Materials and Methods

### 2.1. Murine Model of Hyperthyroidism

Hyperthyroidism was induced in ten-week-old female Balb/c (H-2d) mice by treatment with 12 mg/l of T4 (Sigma-Aldrich, St. Louis, Mo., USA) in the drinking water for 30 days [17]. Animals were housed in a controlled environment (12-hour light-dark cycle, at 18°C with ad libitum access to food and water), at the Instituto de Investigaciones Biomédicas (CONICET, Buenos Aires, Argentina) in accordance with the recommendations in the *Guide for the Care and Use of Laboratory Animals* of the National Institutes of Health (NIH publication No. 85-23, 1996). The protocol used was approved by the Institutional Committee for the Care and Use of Laboratory Animals, Faculty of Medicine, Argentine Catholic University. To confirm the thyroid status, serum levels of T3 and T4 were evaluated using a commercial RIA kit (Immunotech, Prague, Czech Republic) following the supplier's instructions, and TSH serum levels were determined using an ELISA kit (Uscn Life Science Inc., Wuhan, Hubei, China).

### 2.2. *In Vitro* Treatment of Lymphoid Cells

Lymphocytic cells obtained from lymph nodes and spleen according to previously described protocols [2] were resuspended in the RPMI 1640 medium with 10% bovine fetal serum and 2% antibiotics penicillin and streptomycin. The cells were dispensed in 24-well plates at a concentration of 1 × 10^6^ cells/ml and incubated at 37°C, in an atmosphere with 5% CO_2_ pressure. The cells were incubated in the absence or presence of H_2_O_2_ (250 *μ*M) for 4 hours and/or N-acetyl-cysteine (NAC; 2 mM) for 12 hours. Once the incubation periods ended, the cells were collected in Eppendorf tubes, centrifuged at 800xg, and resuspended in phosphate-buffered saline to eliminate the H_2_O_2_ and the NAC not incorporated into the cells. Subsequently, the cells were used to evaluate the production of reactive oxygen species and the genomic expression of the antioxidant enzymes CAT, GPx-1, and SOD-1.

### 2.3. Reactive Oxygen Species Quantification

Lymphocytic cells (1 × 10^6^ cells) obtained from lymph nodes and spleen were resuspended in 1 ml phosphate-buffered saline and incubated with 10 *μ*M DCFH-DA for 30 min at 37°C. Fluorescence intensity of the oxidized probe was measured by a flow cytometer (BD Accuri™, Becton Dickinson Biosciences) at 488 nm and analyzed using the BD Accuri C6 software [18].

### 2.4. Genomic Expression of Antioxidant Enzymes

The messenger RNA expression of antioxidant enzymes was evaluated by reverse transcriptase (RT) followed by conventional polymerase chain reaction (PCR) or real-time PCR amplification. Briefly, total RNA was extracted of 5 × 10^6^ lymphocytic cells using 1 ml TRI Reagent (Genbiotech SRL) per sample, followed by treatment with 200 *μ*l chloroform. The tubes were centrifuged at 12,000xg and the RNA in the aqueous phase was pelleted by the addition of ethanol and subsequent centrifugation at 12,000xg. The RNA pellets were dissolved in RNase-free water, and the RNA concentration was measured spectrophotometry at 260 nm (NanoDrop ND-1000, UK). The cDNA was synthesized by incubation of 2 *μ*g of total RNA with 1 *μ*M of oligodeoxythymidine12-18 (Biodynamics SRL), 5 mM of dNTP (Promega), and 4 units of reverse transcriptase (Omniscript kit, Qiagen), in a total volume of 20 *μ*l, at 37°C for 1 hour. Convencional PCR was performed in a thermal cycler (Progene, Techne) using 5 *μ*l of cDNA (2.5 *μ*g), 1 *μ*l of each primer (20 *μ*M), and 12.5 *μ*l de reaction mixture containing 25 mM MgCl_2_, 25 mM of dNTP, and 1.5 U of Taq DNA polymerase (Biodynamics SRL) and RNAse free-water until reaching a total volume of 25 *μ*l. The conventional PCR products were run on a 2% agarose gel and stained with ethidium bromide. The amplification by qPCR was performed in a Rotor-Gene 6000 using SYBR Green technology (Biodynamics SRL). Genomic expression was quantified by the comparative cycle threshold (Ct) method (Applied Biosystems). The primers (IDT, Biodynamics SRL) used were as follows: 5′-AGTCTTCGTCCCGAGTCTCTC-3′ (CAT forward) and 5′-CTGGTCGGTCTTGTAATGGAA-3′ (CAT reverse), 5′-GGACTACACCGAGATGAACGA-3′ (GPx-1 forward) and 5′-GAGCCTTCTCACCATTCACTTC-3′ (GPx1 reverse), 5′-AGGCTGTACCAGTGCAGGAC-3′ (SOD-1 forward) and 5′-GTTTACTGCGCAATCCCAAT-3′ (SOD-1 reverse), and 5′-GCTATCCAGAAAACCCCTCAA-3′ (*β*2-microglobulin forward) and 5′-CATGTCTCGATCCCAGTAGACGGT-3′ (*β*2-microglobulin reverse). The cycling conditions were 95°C for 5 min and 32 cycles of 95°C for 1 min, 62°C for 1 min (for CAT and GPx-1) or 60°C for 1 min (for SOD-1 and *β*2-microglobulin), and 72°C for 1 min, followed for a final extension at 72°C for 10 min.

### 2.5. Western Blot Analysis

Lymph node and spleen cells of the study groups were resuspended in the RPMI 1640 medium with 10% SFB and were centrifuged and resuspended in lysis buffer (50 mM Tris-HCl, 150 mM NaCl, 1 mM EGTA, 1 mM NaF, 1 mM Na_3_VO_4_, 1 *μ*M phenylmethylsulfonyl fluoride, 0.25% sodium deoxycholate, 1% Igepal, and 10 *μ*M of protease inhibitors (aprotinin, pepstatin, and leupeptin)) for 20 min at 4°C. Protein extracts were resuspended in SDS sample buffer (2% SDS, 10% glycerol, 62.5 mM Tris-HCl, pH 6.8, 0.2% bromophenol blue, and 1% 2-mercaptoethanol), then separated by SDS-PAGE, and transferred to nitrocellulose membranes. Nitrocellulose membranes were blocked with blocking buffer (5% nonfat dried milk dissolved in 100 mM Tris-HCl with 0.1% Tween-20, pH 7.5) for 1 h at room temperature. Then, nitrocellulose membranes were incubated overnight with mouse anti-catalase antibody (1 : 1000, Sigma-Aldrich), rabbit anti-gluthathione peroxidase-1 (1 : 1000, Abcam), rabbit anti-superoxide dismutase-1 (1 : 1000, Abcam), rabbit anti-phospho-Nrf-2 antibody (1 : 1000, Thermo Fisher Scientific, USA), rabbit anti-PKC*α*, PKC*β* (1 : 1000, Sigma-Aldrich) or rabbit anti-PKC*γ* (1 : 1000, Santa Cruz Biotechnology Inc.), mouse anti-phosphorylated ERK 1/2 (1 : 1000, Santa Cruz Biotechnology) or rabbit anti-total ERK (1 : 1000, Santa Cruz Biotechnology), and rabbit anti-*β*-tubulin antibody (1 : 2000, Santa Cruz Biotechnology). Membranes were then incubated with a goat anti-rabbit horseradish peroxidase-conjugated antibody (1 : 2500, Abcam) for 1 h, and the specific bands were revealed by chemiluminescence (ECL Plus, GE Healthcare) using the ImageQuant LAS 4000 digital imaging system (GE Healthcare, UK). Densitometric analysis was performed with the ImageJ software (version 5.1, Silk Scientific Corporation, NIH, Bethesda, MA, USA).

### 2.6. Immunocytochemistry and Confocal Microscopy

For immunocytochemical analysis, lymphocyte cells of euthyroid and hyperthyroid mice were seeded in microscopy slides and fixed with 3.7% formaldehyde for 15 min. The cells were washed with PBS, permeabilized with 0.2% Triton X-100 for 10 min, and then blocked with PBS-BSA (1%) for 1 h, at room temperature (RT). The anti-Nrf2 antibody (dilution 1 : 500; Thermo Fisher Scientific) was added, and the slides were incubated for 2 h at RT. After removal of the primary antibody, FITC-labeled secondary antibody (dilution 1 : 1500; Abcam) was added and the slides were incubated in a dark humidified chamber for 2 h. The nuclei were marked with propidium iodide (PI; 10 *μ*g/ml). Images were acquired with a confocal LSM Zeiss 510.

### 2.7. PKC Activity Quantification

Lymphocytic cells obtained from lymph nodes and spleen were frozen at -70°C. Where it is indicated, the cells were treated with staurosporine (5 nM) for 2 hours prior to freezing. PKC activity was evaluated in lymphoid cells (10 × 10^6^ cells/sample) by measuring the incorporation of ^32^P from [*γ*-^32^P]-ATP into histone H1 [19]. Briefly, the cell extracts were incubated for 30 min at 37°C with a reaction mixture that contained 25 mM ATP (0.4 mCi), 50 mg histone H1, 10 mg/ml of phosphatidylserine vesicles, 0.2 mM CaCl_2_, 10 mM magnesium acetate, 5 mM *β*-mercaptoethanol, and 20 mM HEPES, pH 7.5, in a final volume of 85 *μ*l. The enzymatic activity was stopped by the addition of 5% trichloroacetic acid with 10 mM H_3_PO_4_. The reaction mixture was filtered using GF/C glass fiber filters, and the radioactivity incorporated into histone H1 retained in the filters was quantified in a scintillation counter. The PKC activity was expressed as picomoles of ^32^P incorporated into the histone H1 per minute and per 10^7^ cells.

### 2.8. Statistic Analysis

The results are expressed as mean ± standard error. The statistical differences between the means of the two study groups were analyzed using the GraphPad Prism version 4.0 sofware; two-way ANOVA followed by Tukey's *post hoc* analysis was used to assess statistical significance.

## 3. Results

### 3.1. Development of the Murine Model of Hyperthyroidism and Evaluation of Treatment Effectiveness

**Figure 1:**
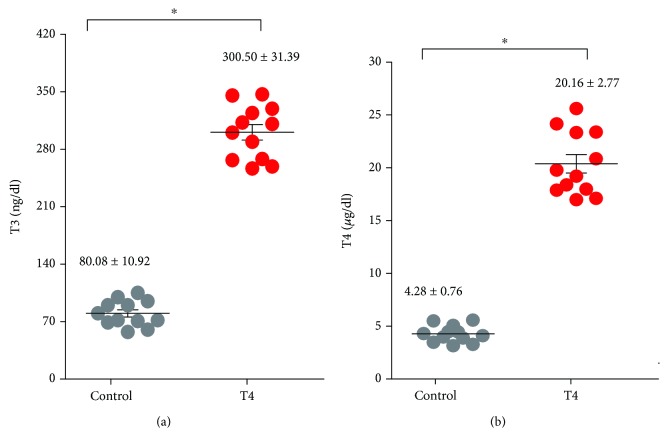
Evaluation of serum levels of thyroid hormones. The levels of T3 and T4 were determined by radioimmunoassay in serum samples from control mice (euthyroid) or treated with T4 (hyperthyroid), as described in Materials and Methods. The average values of each experimental group ± ES are shown in parentheses. ∗ differs significantly from the control group with *p* < 0.05; *n* = 12 mice were used in each group.

To analyze the effects of thyroid hormones on the modulation of oxidative parameters in lymphocyte tissues, we established murine models of hyperthyroidism in Balb/c mice, by treating them with T4 in the drinking water for 30 days, as detailed in Materials and Methods. To corroborate the thyroid status of the animals after the end of the treatment, we measured thyroid axis hormones in the mouse serum. As expected, the hyperthyroid animals had serum levels of T3 and T4 greater than the euthyroid controls (Figure 1) and significantly lower circulating TSH levels (T3 hyperthyroid: 300.50 ± 31.39 ng/dl versus T3 euthyroid: 80.08 ± 10.92 ng/dl, T4 hyperthyroid: 20.16 ± 2.77 *μ*g/dl versus T4 euthyroid: 4.28 ± 0.76 *μ*g/dl, and TSH hyperthyroid: <20 ng/ml versus TSH euthyroid: 47.8 ± 5.4 ng/ml).

### 3.2. Modulation of the Levels of Reactive Oxygen Species

**Figure 2:**
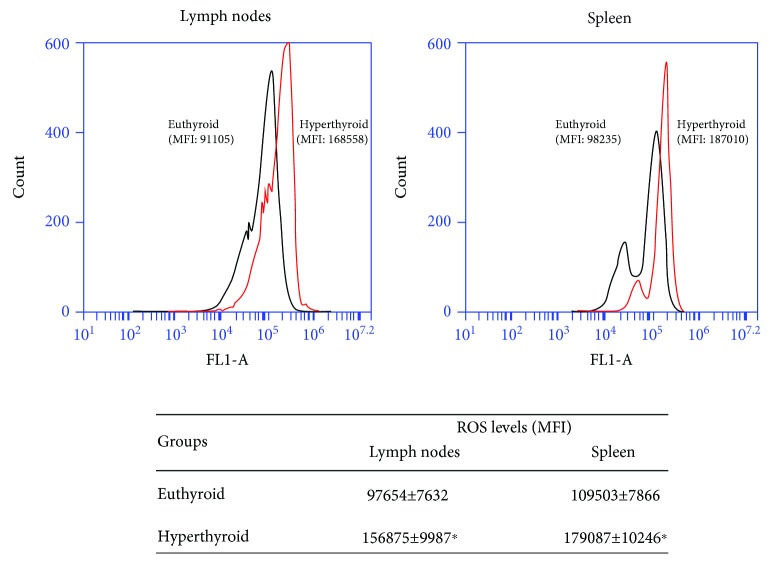
Modulation of the levels of reactive oxygen species. Lymphocyte cells of euthyroid and hyperthyroid mice were incubated with 10 *μ*M DCFH-DA for 30 min at 37°C, and the fluorescence intensity of the oxidized probe was measured by a flow cytometer at 488 nm. The histograms shown are representative of 8 independent experiments performed in duplicate. The average values of mean fluorescence intensity (MFI) ± ES are indicated in the table. ∗ differs significantly from the euthyroid mice with *p* < 0.05.

To evaluate the production of ROS, the lymphocyte cells were obtained from lymph nodes and spleen of euthyroid and hyperthyroid mice and incubated with the DCFH-DA probe. The fluorescence of DCF-DA oxidized by oxygen free radicals (SO^2-^, H_2_O_2_, and OH^·^) is indicative of the total levels of intracellular ROS. As shown in Figure 2, the lymphoid cells of the lymph node and spleen obtained from the hyperthyroid mice showed a higher production of ROS with respect to the cells of euthyroid mice.

### 3.3. Expression of Antioxidant Enzymes

**Figure 3:**
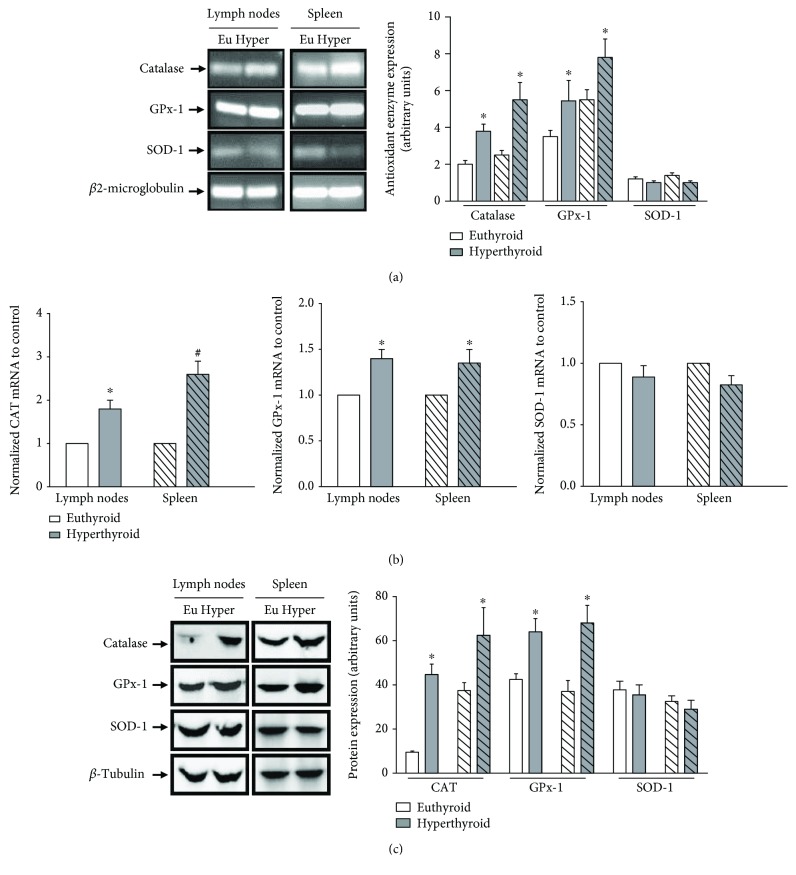
Genomic and protein expression of antioxidant enzymes. (a) The genomic expression of CAT, GPX-1, and SOD-1 was evaluated by conventional PCR. The amplified products were subjected to agarose gel electrophoresis and stained with ethidium bromide. Representative gels from 4 independent experiments performed in duplicate are shown. The bar graph shows the densitometric analysis of each band relativized to *β*-microglobulin used as housekeeping. The smooth bars correspond to lymph node samples, and stripe bars correspond to the spleen samples. ∗ differs significantly from euthyroid controls with *p* < 0.05. (b) The genomic expression of the antioxidant enzymes was quantified by real-time PCR (bar graphs). ∗ differs significantly from euthyroid controls with *p* < 0.05 or # differs significantly from euthyroid controls with *p* < 0.01. (c) The protein expression of CAT (60 kDa), GPX-1 (22 kDa), and SOD (32 kDa) was determined by western blot assays from cell extracts obtained from lymph nodes and spleens of euthyroid and hyperthyroid mice. Representative immunoblots from 4 independent experiments are shown. The bar graph shows the densitometric analysis of each band relativized to *β*-tubulin used as housekeeping. ∗ differs significantly from the lymphoid cells of euthyroid mice with *p* < 0.05.

**Figure 4:**
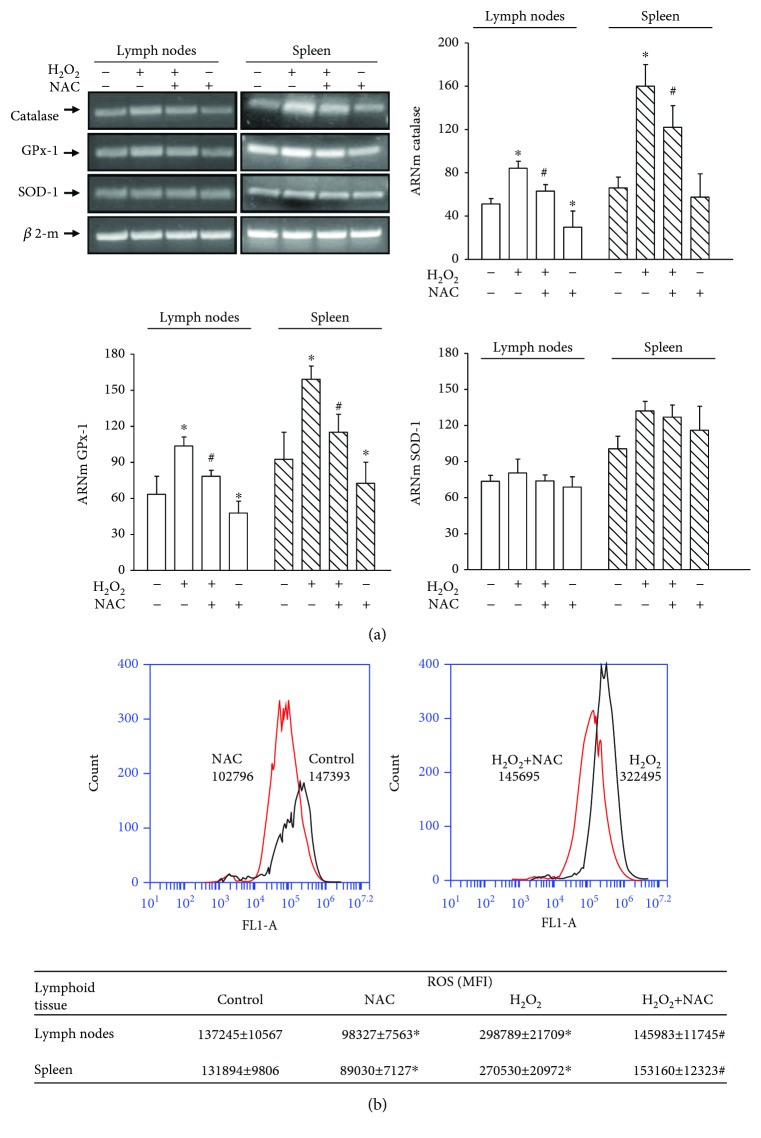
Induction of ROS on the genomic expression of antioxidant enzymes. (a) Lymph nodes and spleen cells of euthyroid mice were incubated at 37°C in the absence or presence of H_2_O_2_ (250 *μ*M) for 4 hours and/or N-acetyl-cysteine (NAC; 2 mM) for 12 hours. Then, the genomic expression of CAT, GPX-1, and SOD-1 was evaluated by conventional PCR. The amplified products were subjected to agarose gel electrophoresis and stained with ethidium bromide. Representative gels from 3 independent experiments performed in duplicate are shown. The bar graph shows the densitometric analysis of each band relativized to *β*-microglobulin used as housekeeping. ∗ differs significantly from control cells without treatment with *p* < 0.05; # differs significantly from cells treated with H_2_O_2_ with *p* < 0.05. (b) The lymph node and spleen cells were treated as described above, and then, the cells were incubated with 10 *μ*M DCFH-DA to evaluate the production of reactive oxygen species by a flow cytometer. Histograms show the production of ROS in lymph node cells subjected to different treatments. Similar results were obtained in spleen cells (data not shown). The average values of mean fluorescence intensity (MFI) ± ES are indicated in the table. The results shown are representative of 3 independent experiments performed in duplicate. ∗ differs significantly from control cells without treatment with *p* < 0.05; # differs significantly from cells treated with H_2_O_2_ with *p* < 0.05.

The increase in ROS production mediated by hyperthyroidism was correlated with an increase in the genomic expression of the antioxidant enzymes CAT and GPx-1 in lymphoid cells of lymph nodes and spleen, evaluated by conventional PCR and real-time PCR (Figure 3(a)). The increase in the genomic expression of CAT and GPx-1 in lymphoid cells of hyperthyroid mice resulted in an increase in the protein expression of these enzymes (Figure 3(b)). We did not find significant differences in the genomic and protein expression of SOD-1 between both study groups. To elucidate whether the increase in the genomic and protein expression of CAT and GPx-1 was a direct consequence of the exacerbated increase of oxidative species or a direct stimulatory action of thyroid hormones in the expression of antioxidant enzymes, we decided to incubate *in vitro* lymphocytes of euthyroid mice in the presence of an exogenous inductor of oxidative stress (H_2_O_2_, 250 *μ*M) and analyzed the genomic expression of the antioxidant enzymes by the conventional PCR technique. As shown in Figure 4(a), lymphoid cells of lymph nodes and spleen incubated for 4 hours with 250 *μ*M H_2_O_2_ showed an increase in the expression of mRNA of the CAT and GPx-1 enzymes. The pretreatment of these cells for 12 hours with the antioxidant N-acetyl-cysteine (NAC, 2 mM) before the addition of H_2_O_2_ inhibited the increase in gene expression of antioxidant enzymes, as well as decreased levels of gene expression in the control cells without treatment. The addition of H_2_O_2_ to the cultures did not modulate the gene expression of SOD-1. Additionally, we analyzed the levels of ROS produced in the cells subjected to the different culture conditions. As expected, the treatment with exogenous peroxide increased the levels of oxidative stress and the cells preincubated with NAC before the addition of peroxide showed ROS levels similar to those of control cells. Incubation of the cells alone in the presence of NAC induced a significant decrease in ROS levels (Figure 4(b)).

### 3.4. Activation of Nrf-2 Mediated by PKC and ERK Kinases

**Figure 5:**
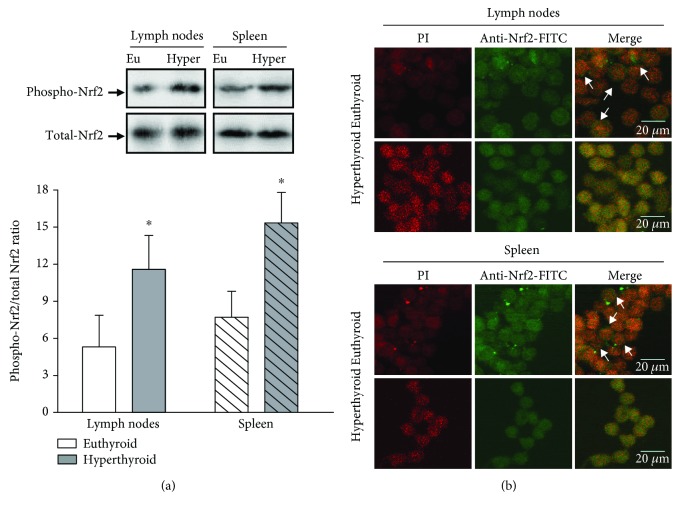
Phosphorylation of Nrf-2. (a) The expression of phosphorylated Nrf-2 and total Nrf-2, in lymph node and spleen cells of euthyroid and hyperthyroid mice, was quantified by western blot assays, using a specific antibody that recognizes the phosphorylation in the amino acid serine 40 of the transcription factor (phosphorylated Nrf-2) or a specific antibody that recognizes the fragment corresponding to a region within amino acids 108 and 413 of the transcription factor (total Nrf-2). Representative immunoblots of 3 independent experiments are shown. The bar graph shows the densitometric analysis of phosphorylated Nrf-2 bands relativized to total Nrf-2. ∗ differs significantly from the lymphoid cells of euthyroid mice with *p* < 0.05. (b) Nrf-2 localization in the cell nucleus was evaluated in lymph node and spleen cells of euthyroid and hyperthyroid mice by immunocytochemical analysis and confocal microscopy as described in Materials and Methods. The photographs are representative of 3 independent assays. The arrows indicate the cytoplasmic region marked in green corresponding to Nrf-2 not translocated to the nucleus.

**Figure 6:**
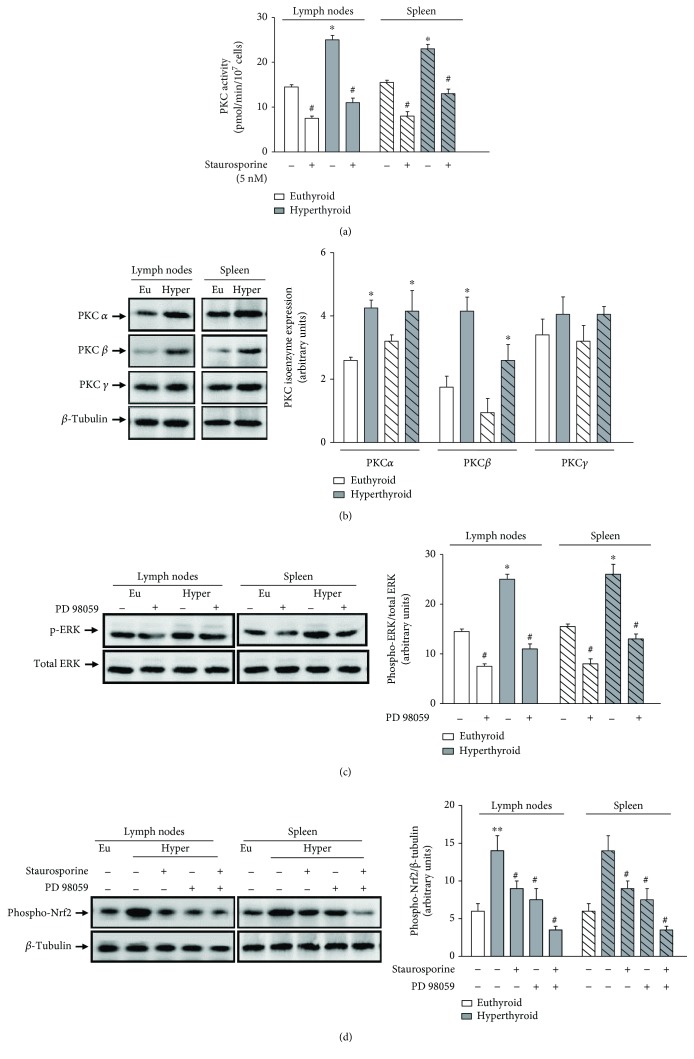
Activation of Nrf-2 mediated by PKC and ERK kinases. (a) Lymph nodes and spleen cells of euthyroid and hyperthyroid mice were incubated in the absence or presence of staurosporine (5 nM) for 2 hours, and then, the PKC activity was evaluated as described in Materials and Methods. The results shown are representative of 4 independent experiments performed in triplicate. ∗ differs significantly from the lymphoid cells of euthyroid mice without treatment with *p* < 0.05. ^#^Cells treated with staurosporine differ significantly from their respective controls with *p* < 0.05. (b) The protein expression of PKC*α* (76.8 kDa), PKC*β* (76.7 kDa), and PKC*γ* (78.4 kDa) was determined by western blot assays from cell extracts obtained from lymph nodes and spleens of euthyroid and hyperthyroid mice. Representative immunoblots from 4 independent experiments are shown. The bar graph shows the densitometric analysis of each band relativized to *β*-tubulin used as housekeeping. The smooth bars correspond to the lymph node samples, and the stripe bars correspond to the spleen samples. ∗ differs significantly from the lymphoid cells of euthyroid mice with *p* < 0.05. (c) Lymph nodes and spleen cells of euthyroid and hyperthyroid mice were incubated in the absence or presence of PD 98059 (20 *μ*M) for 2 hours, and then, the phospho-ERK expression was evaluated by western blot assays from cell extracts obtained from these cells. Representative immunoblots from 3 independent experiments are shown. The bar graph shows the densitometric analysis of each band relativized to total ERK. ∗ differs significantly from the lymphoid cells of euthyroid mice without treatment with *p* < 0.05. ^#^Cells treated with PD 98059 differ significantly from their respective controls with *p* < 0.05. (c) Phosphorylation of Nrf-2 was analyzed by western blot assays from protein extracts obtained from lymphoid tissue cells of hyperthyroid mice, preincubated in the absence or presence of staurosporine (5 nM) or PD 98059 (20 *μ*M) or both inhibitors together. Representative immunoblots from 3 independent experiments are shown. The bar graph shows the densitometric analysis of each band relativized to *β*-tubulin used as housekeeping. ∗ differs significantly from the lymphoid cells of euthyroid mice without treatment with *p* < 0.05. ^#^Cells treated with PKC or ERK inhibitors differ significantly from lymphoid cells of hyperthyroid mice without treatment with *p* < 0.05.

As described, Nrf-2 plays a key role in protecting the cell from oxidative stress by upregulating genes involved in cellular detoxification and antioxidant defense. Therefore, we evaluate the participation of Nrf-2 in the modulation of oxidative stress mediated by hyperthyroidism. First, we analyzed the phosphorylation levels at the amino acid serine 40 of Nrf-2. The results indicate a significant increase in phosphorylation levels of Nrf-2 in lymph node and spleen cells of hyperthyroid mice with respect to euthyroid mice. The increase in phosphorylation levels of Nrf-2 was correlated with the translocation of the nuclear factor from cytosol to the nucleus (Figure 5). Then, we analyze the participation of protein kinase C (PKC) and extracellular signal-regulated kinases (ERK1/2) in the phosphorylation of Nrf-2. As shown in Figure 6(a), the kinase activity of PKC is increased in the lymphoid cells of the hyperthyroid mice with respect to euthyroid control animals. The preincubation of these cells with staurosporine (5 nM), an inhibitor of the PKC activity, decreased the levels of enzymatic activity to values lower than 40% of the total activity, in the absence of staurosporine. The increase in PKC activity in the hyperthyroid mice was correlated with an increase in protein expression of the classical PKC isoenzymes *α*, *β*, and *γ* (Figure 6(b)). Additionally, we found that hyperthyroid conditions increased the levels of phosphorylation of ERK kinase in lymph node and spleen cells. Preincubation of these cells with PD 98059 (20 *μ*M) decreased ERK phosphorylation increment induced by hyperthyroidism, as well as the basal levels of phosphorylated ERK in euthyroid mice (Figure 6(c)).

To verify whether both PKC and ERK kinases were involved in increased phosphorylation of Nrf-2 on hyperthyroid status, we analyzed the level of phosphorylation of Nrf-2 in lymphoid tissue cells of hyperthyroid mice, preincubated in the absence or presence of staurosporine or PD 98059 or both inhibitors together. As shown in Figure 6(d), staurosporine or PD 98059 decreased the phosphorylation levels of Nrf-2 in lymphoid cells of hyperthyroid mice. The inhibition in the phosphorylation of Nrf-2 was even greater when the cells were preincubated in the presence of both inhibitors together, showing levels of phosphorylation of Nrf-2 lower than those found in cells from euthyroid mice (Figure 6(d)).

## 4. Discussion

The aim of this study was to evaluate the effect of TH on the induction of oxidative stress, analyzing the intracellular signals involved in the expression of antioxidant enzymes. We found that hyperthyroidism induced an exacerbated increase of ROS in lymph nodes and spleen of Balb/c mice. Other studies have reported increased levels of oxidative stress mediated by the hyperthyroid state, in different tissues such as testis, skeletal muscle, heart, pancreas, and hippocampus [20–24]. However, oxidative stress has also been reported in hypothyroid conditions [25, 26].

TH exert pleiotropic actions on numerous tissues, being one of their main functions the regulation of cellular metabolism. The overproduction of TH increases energy demand and mitochondrial activity, and as a consequence of the latter, there is an increase in the production of ROS [27]. Several authors have described the presence of T3 receptors in mitochondria [9, 28], whereby the TH could also exert direct actions on mitochondrial activity that contribute to the increase in ROS.

Murine and human lymphoma cells express the membrane receptor of TH (integrin *α*v*β*3) and the *α* and *β* isoforms of the nuclear receptor (TR*α*/*β*). TH exert genomic actions through TR*α*/*β* and nongenomic actions through integrin *α*v*β*3. We have described in lymphoma cells the signaling pathways through these receptors that involve proliferative processes and angiogenesis, and we have shown that TH increase the expression of both receptors [1, 3, 5].

ROS play an essential role in cell signaling pathways and defense mechanisms against pathogens, and their production has been an evolutionarily conserved process. During normal cell metabolism, ROS are produced in physiological concentrations. However, in certain pathological conditions such as inflammatory bowel disease, diabetes, cancer, or obesity, ROS can be produced in excessive amounts leading to apoptosis, necrosis, or autophagy of cells [13].

We found that the increase in the production of ROS in lymphoid cells was correlated with an increase in the genomic and protein expression of antioxidant enzymes CAT and GPx-1.

The gene expression of CAT was greater in spleen cells than in the lymph node cells of hyperthyroid mice. It is possible that the cells of lymph nodes and spleen have different levels of expression of receptors for TH (nuclear or membrane receptors) or other factors involved in the signaling cascade leading to an increased expression of antioxidant enzymes. Previously, we demonstrated the differential expression of nuclear receptors (TRs) or membrane receptors (integrin *α*v*β*3) in different lines of lymphomas, but there are no reports about the level of the expression of TH receptors in normal lymphocytes [1, 3, 5]. In addition, we have shown that hyperthyroidism induces a greater proliferative response to mitogenic stimuli in lymphocytes from the spleen than from the lymph node which could be explained by a differential expression level of TH receptors [2].

Both CAT and GPx-1 enzymes catalyze the detoxification of hydrogen peroxide and lipid peroxides, preserving the intracellular environment in a reduced state and protecting tissues from oxidative damage. CAT is an enzyme with cytosolic distribution that contains iron in its active site, while GPx-1, also of cytosolic distribution, requires selenium as a cofactor. There are three forms of SOD enzyme with diverse locations (mitochondrial, cytosolic, and extracellular fluids), which catalyze the conversion of the superoxide radical to hydrogen peroxide. We evaluated the expression of SOD-1, with catalytic activity in the cytosol and requiring copper and zinc as a cofactor for its enzymatic activity. Although some authors have found that oxidative stress induces the expression of SOD in other tissues [29, 30], we have not found significant differences in the expression of this enzyme, possibly because catalyzing reaction is not favored in the presence of high levels of hydrogen peroxide.

Several studies performed in different tissues show discrepancies about the effect of hyperthyroidism on the expression of antioxidant enzymes. In this sense, Subudhi and Chainy have found a decrease in the genomic expression of SOD1, CAT, GPx1, and glutathione reductase in the liver of hyperthyroid rats and a downregulated CAT gene expression in the cardiac tissue of these animals [31]. Rao et al. had found that T4-induced hyperthyroidism decreases the SOD, CAT, and GPx levels in the hippocampus of an aged female golden hamster [24]. Other studies, in female mice treated with T4, showed a lower gene expression of oxidative enzymes in the mouse ovary [32], while Morini et al. found an increase in GPx and GR activity in the liver of hyperthyroid rats [33] and Messarah et al. had found an increase of SOD, CAT, and GPx activities in the heart and erythrocytes of hyperthyroid rats [8]. The expression of antioxidant enzymes mediated by hyperthyroidism would be dependent on the cell type, the mitochondrial activity, and the intracellular concentration of ROS.

All antioxidant enzymes require mineral cofactors for their functionality. There is evidence that the thyroid state modulates the differential distribution of these minerals [34], regulating their concentration in different tissues. This could affect the enzymatic activity and the regulation of the cellular redox state, but the mineral distribution and its modulation on the enzymatic activity have not yet been analyzed in lymphoid tissues.

To elucidate whether ROS induced the increase in the genomic expression of antioxidant enzymes, we incubated lymphocyte cells in the presence of 250 *μ*M of H_2_O_2_ to induce oxidative stress. We demonstrated that, in the absence of thyroid hormones, ROS lead to the expression of antioxidant enzymes. As expected, pretreatment of these cells with N-acetyl-cysteine, a precursor of reduced glutathione synthesis, prior to H_2_O_2_ treatment, inhibited the increase in gene expression of antioxidant enzymes. These findings are in agreement with other studies that show that the oxidative stress generated in diseases such as diabetes or obesity induces the expression of antioxidant enzymes [35].

Nrf-2 is an important transcription factor which can directly regulate antioxidant proteins expression. Several studies have shown that oxidative stress induces the expression of the cysteine uptake transporter and heme oxygenase-1 through the activation of Nrf-2. These proteins are essential in the regulation of oxidative balance and cell survival [13].

Under conditions of low oxidative stress, Nrf-2 interacts with Kelch-like ECH-associated protein 1 (Keap-1), forming a complex in the cytoplasm susceptible to ubiquitination and degradation in proteasome. But if ROS levels increase in the cell, ROS can oxidize redox-sensitive cysteine residues on Keap-1 and induce the dissolution of the Keap1/Nrf-2 complex. Nrf-2 can then translocate to the nucleus and bind to antioxidant responsive elements (ARE) of many antioxidant genes and regulate their expression [36, 37].

Several authors have described the participation of Nrf-2 in the expression of antioxidant enzymes in other tissues [38], but its role in the activation of the enzymatic antioxidant system in lymphoid cells has not been studied. Therefore, we first proposed to analyze the phosphorylation levels of Nrf-2, using an antibody that specifically recognizes the phosphorylated serine in position 40. We found a significant increase in the phosphorylation of Nrf-2 in lymphoid tissue of hyperthyroid mice.

Posttranslational modifications of Nrf-2 have been described, such as phosphorylations in serine and threonine amino acid residues mediated by various kinases such as phosphatidylinositol 3-kinase (PI3K), the c-Jun N-terminal kinase (JNK), and the p38 protein. Phosphorylation by these enzymes apparently facilitates the dissociation of Nrf2 from Keap1 and its subsequent translocation to the cell nucleus [39]. We had previously demonstrated that PKC and ERK protein kinases were involved in the signaling pathways triggered by TH thus leading to the survival and proliferation of normal and tumor lymphoid cells [2, 3, 40]. So, the participation of these kinases in the phosphorylation of Nrf-2 was of our interest.

We found an increase in the activity of PKC in lymphoid tissue of hyperthyroid mice, associated with the increase in protein expression of the classical isoenzymes of PKC*α*, *β*, and *γ*. These enzymes are expressed in lymphoid cells, and their activity is dependent on Ca^2+^ and phospholipids [19]. The hyperthyroid state of the mice also increased the activity of the ERK kinase.

To demonstrate that both PKC and ERK kinases were involved in the phosphorylation of the transcription factor Nrf-2, we analyzed the level of phosphorylation of Nrf-2 in lymphoid tissue cells from hyperthyroid mice, preincubated in the absence or presence of staurosporine or PD 98059 or both. Staurosporine is a potent, cell-permeable, and broad spectrum inhibitor of protein kinases, such as protein kinase C (IC50 = 3 nM), cAMP-dependent protein kinase (IC50 = 8 nM), and p60v-src (IC50 = 6 nM). We use a concentration of 5 nM of staurosporine to ensure that PKC activity decreases to values below 50% and the other two kinases are affected to a lesser extent. The concentration of PD 98059 (20 *μ*M) used had been previously tested in the previous study [3]. The induction of Nrf-2 phosphorylation mediated by the hyperthyroid state was significantly decreased in the presence of staurosporine or PD 98059. The inhibition in the phosphorylation of Nrf-2 was even greater when the cells were preincubated in the presence of both inhibitors together. These results demonstrate the participation of PKC and ERK kinases in the phosphorylation of the transcription factor Nrf-2.

Although, we have shown that ROS by themselves can induce the genomic expression of antioxidant enzymes, the presence of TH that stimulate signaling pathways that lead to increased basal activity of ERK and PKC kinases is necessary. PKC and ERK kinases phosphorylate Nrf-2, facilitating its translocation to the cell nucleus, increasing the transcriptional activity of antioxidant enzymes, and thus contributing to the balance of the cellular redox state.

In most tissues, cells are exposed to frequent changes in levels of oxidative stress and inflammation. Nrf-2 and NF-*κ*B are the two key transcription factors that regulate cellular responses to oxidative stress and inflammation, respectively. Several studies suggest that there is functional cross talk between these two pathways. The absence of Nrf-2 can exacerbate NF-*κ*B activity leading to increased cytokine production, whereas NF-*κ*B can modulate the transcription and activity of Nrf-2 [41]. We previously demonstrated that the TH increase the activity of PKC in tumor lymphoid cells and that this enzyme induce the translocation of NF-*κ*B from cytosol to the nucleus [40, 41]. In addition, our studies show the phosphorylation of Nrf-2 mediated by the PKC enzyme. The cross talk between NF-*κ*B and Nrf-2 signaling pathways could regulate the expression of antioxidant enzymes and inflammation, maintaining cellular homeostasis.

**Scheme 1:**
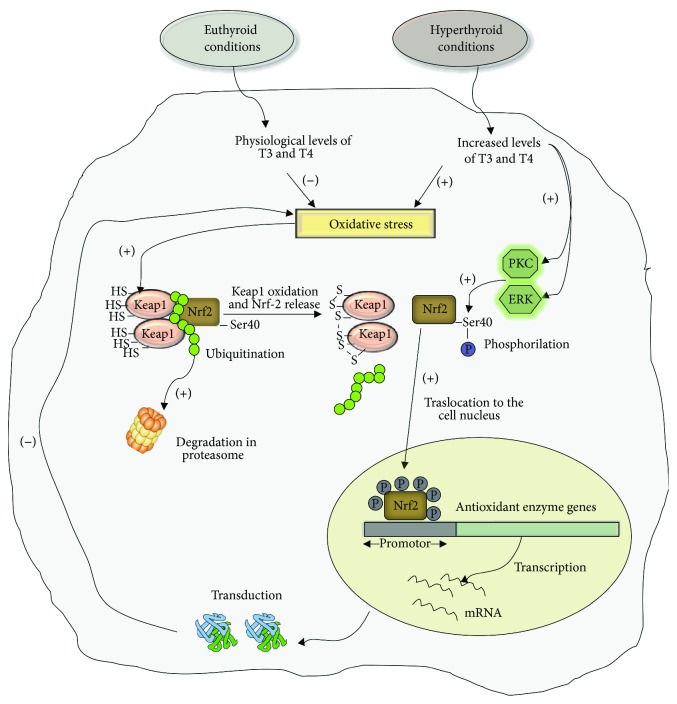
Transduction signals in lymphocytes involved in the regulation of oxidative stress induced by hyperthyroidism. Hyperthyroidism is characterized by increased levels of circulating T3 and T4. The excess of thyroid hormones in lymphoid cells induces an increase in oxidative stress that leads to the oxidation of numerous proteins, including Keap-1. Under euthyroid conditions the Keap-1 dimer binds to Nrf-2. This interaction induces the ubiquitination of Nrf-2 and its consequent degradation in the proteasome; therefore, the levels of this factor are diminished in the cytoplasm. However, under hyperthyroid conditions, the reactive oxygen species oxidize the sulfhydryl groups of Keap-1/Nrf-2 complex. In this way, free Nrf-2 can be phosphorylated by PKC and ERK, two kinases that increase their activity in the hyperthyroid state. Phosphorylated Nrf-2 translocates to the cell nucleus and activates the transcription of antioxidant enzyme genes. The increased expression of antioxidant enzymes contributes to diminish the levels of oxidative stress generated by hyperthyroid conditions.

The regulation of the cellular redox state is essential to avoid the deleterious effect of free radicals on the viability or functionality of lymphocyte cells. We describe the molecular mechanisms by which hyperthyroidism regulates the cellular enzymatic antioxidant system in lymphoid cells (Scheme 1). The knowledge of these molecular mechanisms could establish new molecular targets for more effective therapies in the treatment of patients who have pathologies associated with high levels of oxidative stress, such as obesity or diabetes.

## Data Availability

The data used to support the findings of this study are available from the corresponding author upon request (mlbarreiro@yahoo.com.ar).
